# Demographic Predictors of Mothers' Willingness to Vaccinate Young Children Against COVID-19, Get Tested and Isolate: A Cross-Sectional Survey Before and During the Greater Sydney Lockdown 2021, Australia

**DOI:** 10.3389/fpubh.2022.904495

**Published:** 2022-05-27

**Authors:** Li Ming Wen, Huilan Xu, Chris Rissel, Erin Kerr, Limin Buchanan, Sarah Taki, Philayrath Phongsavan, Reuel Kangjie Chua, Myna Hua, Karen Wardle, Lisa Simone, Alison Hayes, Louise A. Baur

**Affiliations:** ^1^Health Promotion Unit, Population Health Research & Evaluation Hub, Sydney Local Health District, Sydney, NSW, Australia; ^2^Sydney School of Public Health, Faculty of Medicine and Health, The University of Sydney, Sydney, NSW, Australia; ^3^NHMRC Centre of Research Excellence in the Early Prevention of Obesity in Childhood (EPOCH), Sydney, NSW, Australia; ^4^Sydney Institute for Women, Children and Their Families, Sydney Local Health District, Sydney, NSW, Australia; ^5^Charles Perkins Centre, The University of Sydney, Sydney, NSW, Australia; ^6^Health Promotion Service, Population Health, South Eastern Sydney Local Health District, Sydney, NSW, Australia; ^7^Health Promotion Service, Population Health, South Western Sydney Local Health District, Sydney, NSW, Australia; ^8^Discipline of Child and Adolescent Health, Sydney Medical School, The University of Sydney, Sydney, NSW, Australia

**Keywords:** COVID-19, 2019 corona virus disease, a cross-sectional survey, young children, vaccination, health promotion

## Abstract

**Background and Objectives:**

Having a COVID-19 vaccination, getting tested, and self-isolating if symptomatic are some of the most important mitigation strategies for preventing the spread of COVID-19. This study aimed to investigate whether demographic factors are associated with mothers' willingness to vaccinate their 4-year-old children against COVID-19 if a suitable vaccine becomes available or to get tested and self-isolate if they themselves have COVID-19 symptoms and whether the willingness could be influenced by the Greater Sydney lockdown 2021.

**Methods:**

A cross-sectional telephone survey was conducted between 24th February and 26th October 2021. Questions from the NSW Adult Population Health Survey and from previously published studies were used to assess family demographics, mothers' willingness to vaccinate their young children, and willingness to get tested and self-isolate if symptomatic. The survey involved 604 mothers of children aged 4 years who participated in an existing trial in Sydney, Australia.

**Results:**

Mothers were more willing to vaccinate their children when the child's father had a tertiary education or higher, with an adjusted odds ratio (AOR) of 2.60 (95% CI 1.67–4.04). Mothers who were older than 30 years or who completed the survey during the lockdown were more willing to get tested if symptomatic, with AOR 2.50 (95% CI 1.17–5.36) and AOR 3.36 (95% CI 1.41–8.02), respectively. Mothers who were married or had de-facto partners were more willing to self-isolate if symptomatic [AOR 17.15 (95% CI 3.56–82.65)].

**Conclusion:**

Fathers' educational level, mothers' age, and marital status were associated with mothers' willingness to vaccinate their young children if a suitable vaccine were available, to get tested, and self-isolate if symptomatic respectively. The promotion of mitigation strategies for tackling the COVID-19 pandemic needs to take into account specific family demographics.

## Introduction

Since the beginning of 2022, the COVID-19 pandemic continues with the Omicron variant. It has had a significant impact on mortality and morbidity with over five million deaths recorded worldwide (1). In Australia, as of 19 January 2022 (the time of writing), a total of 1,506,602 cases of COVID-19 have been reported, including 2,841 deaths, and approximately 550,014 active cases (2). Therefore, the development of appropriate public health prevention measures involving all community sectors is paramount for tackling the COVID-19 pandemic.

Prior to the high uptake of vaccination in Australia, the transmission of COVID-19 was managed through strict border closures, lockdowns (i.e., stay at home orders), and high levels of testing and self-isolation in patients who were found to be symptomatic. Vaccinating the population against COVID-19 is widely recognized as a critical measure in preventing hospitalization and deaths caused by the COVID-19 infection. In Australia, COVID-19 vaccinations are available for adults and children older than 5 years (3). Since mid-January 2021, in New South Wales (NSW), 95% of the eligible population aged ≤ 16 years have received two doses of a COVID-19 vaccine, and 78% of children aged 12–15 years (3). The vaccine for children aged 5–11 years became available within NSW on 10 January 2022.

Vaccine hesitancy - delay in acceptance or refusal of available vaccination - is the main barrier to vaccine uptake (4). Recent systematic reviews have identified multiple sociodemographic factors associated with vaccine hesitancy, including being female, lower education, non-white ethnicity, and younger age (5, 6). Other factors related to vaccine hesitancy include concerns about vaccine safety, side effects, and efficacy, as well as mistrust of science and governments (5, 6).

Currently, COVID-19 vaccinations for children aged 0–5 years are not approved. To date, generally speaking, children and adolescents have a low probability of experiencing severe health outcomes and have low mortality rates (7). However, if transmission increases in children, the number of adverse cases will inevitably increase. Understanding family demographic factors associated with mothers' willingness to vaccinate their young children is necessary for the future development of public health campaigns and policies related to preventing COVID-19 in this age group.

In addition, testing and self-isolation when symptomatic, together with healthy hygiene practices, social distancing, and wearing face masks are the most important strategies to reduce COVID-19 transmission. Self-isolation at home has been recommended for those who have tested positive for COVID-19 and those who suspect that they have been infected. Testing can help people determine if they are infected with COVID-19 and whether they are at risk of spreading the infection to others.

There is limited research on factors influencing Australian mothers' willingness to vaccinate their young children and implement the mitigation practices for COVID-19. We conducted this cross-sectional survey with the goal of informing the development of appropriate public health prevention measures that can be tailored to specific family demographics for tackling the COVID-19 pandemic. Specifically, this study aimed to investigate whether family demographic factors were associated with mothers' willingness to vaccinate their 4-year-old children against COVID-19 if a suitable vaccine becomes available; or their willingness to self-isolate and get tested if they have COVID-19 symptoms and whether their willingness was associated with the timing of the Greater Sydney lockdown of 2021.

## Methods

### Study Design

We conducted a cross-sectional telephone survey with mothers of 4-year-old children in Sydney, Australia between 24 February and 26 October 2021. During this survey period, the second wave of the COVID-19 outbreak in early June 2021 covered the period before and during the Greater Sydney lockdown. From 26 June to 10 October 2021, people living the Greater Sydney region experienced another large-scale strict lockdown.

### Survey Participants

The survey participants were mothers of young children aged 4 years who have participated in an existing trial since 2017 (8, 9). Briefly, the mothers and children were originally recruited for a trial aiming to investigate the effectiveness of early childhood obesity prevention using telephone support or text messages. The recruitment process and first 2-year outcomes of the original trial have been reported elsewhere (10–12). The study participants were recruited from four local health districts in NSW. This particular survey was part of the 4th year follow-up study of mothers who remained in the existing trial. Participants provided written or verbal consent before commencing the survey.

### Data Collection and Study Variables

The survey was conducted by a marketing survey company using Computer Assisted Telephone Interviewing (CATI) to collect data from mothers when their children reached 4 years. The data collection period was from 25 February to 26 October 2021. A study variable 'survey timing' (before/during/after the lockdown) was generated according to whether the survey was conducted before or after the NSW Government announcement of a Greater Sydney lockdown from 26 June to 11 October 2021. Family demographics data were extracted from the baseline survey, where we used questions from the NSW Adult Population Health Survey (13). All family demographic data were categorized into groups (see **Table 2**). We used the age of 30 years old as a cut point for the mothers' age group as the early 30s was considered an ideal age to give birth to a child and mothers in their 30s were considered more mature and secure in careers and relationships.

### Study Outcome Variables

We used survey questions based on previously published studies to assess mothers' willingness to vaccinate their young children (14), and willingness to get tested and self-isolate if symptomatic (15). Table 1 shows the survey questions asked and response options provided with coding frames.

**Table 1:** Survey questions, response options and coding frames on willingness to vaccinate their 4-year-child against COVID-19, or to get tested and isolate if symptomatic.

**Study outcome factors**	**Questions**	**Responses**	**Coding**
Willingness to get COVID-19	Currently the COVID-19 vaccines are allowed only for the	□Yes, definitely	1—Yes
vaccine for their child	adults and older teens. IF COVID-19 vaccine becomes	□Unsure but leaning toward yes	
	available for children in the future, would you accept	□Unsure but leaning toward no	0—No
	vaccine for your child/children?	□No, definitely not	
Willingness to get tested if	Do you plan to get tested if you have COVID-19	□Strongly disagree	0—Disagree
symptomatic	symptoms (cough, sore throat, fever)?	□Disagree	
		□Neither agree nor disagree	
		□Agree	1—Agree
		□Strongly agree	
Willingness to isolate at	Do you plan to stay home if you have COVID-19	□Strongly disagree	0—Disagree
home if symptomatic	symptoms (cough, sore throat, fever)?	□Disagree	
		□Neither agree nor disagree	
		□Agree	1—Agree
		□Strongly agree	

To assess mothers' willingness to vaccinate their young children (14), the mother was asked “*Currently the COVID-19 vaccines are allowed only for adults and older teens. If COVID-19 vaccine becomes available for children in the future, would you accept the vaccine for your child/children?*” The options were “*Yes, definitely,” “Unsure but leaning toward yes,” “Unsure but leaning toward no,” and “No, definitely not.”* Further, the answers “*Yes, definitely”* and “*Unsure but leaning toward yes”* were categorized as “*Yes,”* and the answers “*Unsure but leaning toward no”* and “*No, definitely not” were categorized* as “No.”

To assess mothers' willingness to be tested and self-isolate if symptomatic (15), the mother was asked how strongly she agreed with the statements “*I plan to get tested if I have COVID-19 symptoms (cough, sore throat, fever)*” and “*I plan to stay home if I have COVID-19 symptoms (cough, sore throat, fever)*”. A 5-point Likert Scale was used for their response options from “*Strongly agree”* to “*Strongly disagree.”* The answers were dichotomized to “Agree” if the mother answered “*Strongly agree*” or “*Agree,”* and “Disagree” if the mother answered “*Neither agree nor disagree,”* “*Disagree,”* or “*Strongly disagree*.”

### Statistical Analysis

Statistical analyses were undertaken using STATA 16 (StataCorp 2016). All *p*-values were two sided and statistical significance was set at the 5% level. Descriptive analysis was conducted to describe family demographic characteristics for those who completed the 4th year survey and COVID-19 related study outcomes. Pearson's Chi-squared tests were conducted to examine the differences in family demographic characteristics and COVID-19 related study outcomes among those who completed the 4-year survey before and during the lockdown. Numbers and percentages were reported. Fisher's exact test was used when the cells in the contingency tables have expected frequencies of <5.

To investigate whether family demographic factors and the greater Sydney lockdown were related to COVID-19 related outcomes, we built three multiple logistic regression models for willingness to vaccinate their young children; willingness to get tested; and willingness to isolate if symptomatic. A study variable, “survey timing” (before/during the lockdown) was included in all three multiple logistic regression models regardless of its significance as it was the main study factor of interest. All multivariable models were adjusted for group allocation of the existing trial. To investigate which family demographic factors were related to the COVID-19 related study outcomes, we used a backward elimination approach, all family demographic variables that were significant in Pearson's Chi-squared tests or Fisher's exact tests with *p* < 0.25 were entered in the multiple logistic regression models. The least significant variables were progressively dropped until only those with *p* < 0.05 remained. Subsequently, the variables that were dropped were added to the model one at a time to see whether they were significant or confounders to those already in the model. Adjusted odds ratios (AORs) with 95% confidence intervals (CI) were then calculated as a measure of the association.

### Ethics Approval

The ethical approval for this survey was obtained from the Ethics Review Committee of Sydney Local Health District (Protocol No. X16–0360 & LNR/16/RPAH/495 and Protocol No X18–0387 & HREC/18/RPAH/545, 9.17/NOV20).

## Results

Of 604 participating mothers who remained in the trial, 491 mothers completed the COVID-19 survey questions at 4 years of child age, with a response rate of 81%, with 251, 234, and six mothers being surveyed before, during, and after the lockdown, respectively. For the purpose of this study, we combined during and after the lockdown in one group (*n* = 240). There were no statistically significant differences in most family demographic factors between those who did and did not complete the 4-year survey except for age. Table 2 shows the distribution of family demographics of the mothers who completed the survey and their group allocation in the existing trial. There were no statistically significant differences in family demographic characteristics and their group allocation between those who completed the survey before and during the lockdown.

**Table 2:** Family demographics of the mothers who completed the survey and their group allocation in the existing trial in Sydney 2021.

**Family demographics**	**Total**	**Survey before/during lockdown**	
		**Before**	**During**
	***n* (%)**	***n* (%)**	***n* (%)**
**Age (years)**			
<30	122 (25)	63 (25)	59 (25)
≥30	369 (75)	188 (75)	181 (75)
**Country of birth**			
Australia	179 (36)	89 (35)	90 (38)
Overseas	312 (64)	162 (65)	150 (62)
**Language spoken at home**			
English	263 (54)	131 (52)	132 (55)
Other	228 (46)	120 (48)	108 (45)
**Annual household income**			
< $80,000	160 (36)	85 (37)	75 (34)
≥$80,000	290 (64)	142 (63)	148 (66)
**Marital status**			
Married/de-facto partner	465 (95)	241 (96)	224 (93)
Other	25 (5)	9 (4)	16 (7)
**Employment status**			
Employed	333 (68)	173 (69)	160 (67)
Other	158 (32)	78 (31)	80 (33)
**Education level**			
University & Higher	348 (71)	184 (73)	164 (69)
Up to HSC/TAFE	142 (29)	67 (27)	75 (31)
**Fathers' employment status**			
Employed	439 (91)	221 (91)	218 (92)
Other	42 (9)	23 (9)	19 (8)
**Fathers' education level**			
University & Higher	298 (63)	155 (64)	143 (61)
Up to HSC/TAFE	178 (37)	88 (36)	90 (39)
**Intervention allocation**			
Tel-support + SMS	231 (47)	106 (42)	125 (52)
Control	260 (53)	145 (58)	115 (48)

*HSC, Higher School Certificate (Year 12); TAFE, Technical and Further Education*.

*Sample is not necessarily 491 due to missing values*.

Table 3 shows that the majority of mothers were willing to get tested (93%) and self-isolate at home (98%) if they had COVID-19 symptoms. It also shows significantly more mothers strongly agreed to get tested if symptomatic when they completed the survey during the lockdown. The willingness to isolate was similar among those who completed the survey before and during the lockdown. Only 50% of the mothers said “*yes, definitely”* to the question about accepting the vaccine for their children if a suitable vaccine became available.

**Table 3:** Mothers' responses to COVID-19 related study outcomes and survey conducted before and during the Sydney lockdown 2021.

**Covid-19 related study outcomes**	**Total *n* (%)**	**Survey timing**	
		**Before**	**During**
		***n* (%)**	***n* (%)**
**Plan to get tested if symptomatic**			
Strongly agree	316 (64)	156 (62)	160 (67)
Agree	144 (29)	71 (28)	73 (30)
Neither agree nor disagree	17 (4)	14 (6)	3 (1)
Disagree	6 (1)	2 (1)	4 (2)
Strongly disagree	8 (2)	8 (3)	0 (0)
**Plan to isolate at home if symptomatic**			
Strongly agree	361 (73)	186 (74)	175 (73)
Agree	122 (25)	59 (23.6)	63 (26)
Neither agree nor disagree	3 (0.8)	3 (1.2)	0 (0)
Disagree	4 (1)	2 (0.8)	2 (1)
Strongly disagree	1 (0.2)	1 (0.4)	0 (0)
**COVID-19 vaccine acceptance for child**			
Yes, definitely	244 (50)	115 (46)	129 (54)
Unsure but leaning to yes	135 (27)	74 (30)	61 (25)
Unsure but leaning to no	72 (15)	36 (14)	36 (15)
No, definitely not	40 (8)	26 (10)	14 (6)

Tables 4, 5 show the associations of family demographic and lockdown factors with mothers' willingness to vaccinate their young children against COVID-19, or to self-isolate and get tested if symptomatic on bivariate and multivariate analyses, respectively. Table 5 shows that mothers who were older than 30 years and completed the survey during the lockdown were more likely to get tested if symptomatic, with AOR 2.50 (95% CI 1.17–5.36; *p* = 0.02) and AOR 3.36 (95% CI 1.41–8.02; *p* = 0.006), respectively. Compared to unmarried mothers, mothers who were married or had a de-facto partner were more likely to self-isolate if symptomatic with AOR 17.15 (95% CI 3.56–82.65; *p* < 0.001). Mothers were more likely to accept a COVID-19 vaccine for their children if the child's father had a tertiary education or higher, with AOR 2.60 (95% CI 1.67–4.04; *p* < 0.001).

**Table 4:** Associations of family demographic and lockdown factors with mothers' willingness to be isolated and tested if symptomatic, or to vaccinate their young children against COVID-19 on bivariate analyses.

**Variables**	**Total**	**Agree to get tested**		**Agree to self-isolate**		**Accept Covid-19 vaccine**	
		**if symptomatic**		**if symptomatic**		**for children**	
	***n* (%)**	***n* (%)**	***p*-Value**	***n* (%)**	***p*-Value**	***n* (%)**	***p*-Value**
**Age (years)**
<30	122 (25)	109 (89)	0.02	119 (98)	0.42^*^	87 (71)	0.07
≥30	369 (75)	351 (95)		364 (99)		292 (77)	
**Country of birth**
Australia	179 (36)	168 (94)	0.91	176 (98)	1.00^*^	131 (73)	0.11
Overseas	312 (64)	292 (94)		307 (98)		248 (79)	
**Language spoken at home**
English	263 (54)	246 (94)	0.88	259 (98)	1.00^*^	207 (79)	0.39
Other	228 (46)	214 (94)		224 (98)		172 (75)	
**Annual household income**
< $ 80,000	160 (36)	148 (93)	0.41	156 (97.5)	0.06^*^	117 (73)	0.08
≥$ 80,000	290 (64)	274 (94)		289 (99.7)		233 (80)	
**Marital status**
Married/de-facto partner	465 (95)	434 (93)	0.39^*^	460 (99)	0.006^*^	363 (78)	0.04
Other	25 (5)	25 (100)		22 (88)		15 (60)	
**Employment status**
Employed	333 (68)	316 (95)	0.11	330 (99)	0.12^*^	258 (77)	0.83
Other	158 (32)	144 (91)		153 (97)		121 (77)	
**Education level**
University & Higher	348 (71)	329 (95)	0.22	345 (99)	0.05^*^	279 (80)	0.02
Up to HSC/TAFE	142 (29)	130 (92)		137 (96)		100 (70)	
**Father employment status**
Employed	439 (91)	413 (94)	0.32^*^	434 (99)	0.03^*^	338 (77)	0.91
Other	42 (9)	38 (90)		39 (93)		32 (76)	
**Father education level**
University & Higher	298 (63)	283 (95)	0.14	296 (99)	0.06	249 (84)	<0.001
Up to HSC/TAFE	178 (37)	163 (92)		172 (97)		119 (67)	
**Survey timing**
Before lockdown	251 (51)	227 (90)	0.002	245 (98)	0.29	189 (75)	0.31
During lockdown	240 (49)	233 (97)		238 (99)		190 (79)	
**Intervention allocation**
Tel-support + SMS	231 (47)	221 (96)	0.09	227 (98)	1.00^*^	176 (76)	0.62
Control	260 (53)	239 (92)		256 (98)		203 (78)	

* *p-value of Fisher's exact test*.

*HSC, Higher School Certificate (Year 12); TAFE, Technical and Further Education*.

*Sample is not necessarily 491 due to missing values*.

**Table 5:** Associations of family demographic and lockdown factors with mothers' willingness to be isolated and tested if symptomatic, or to vaccinate their young children against COVID-19 on multivariate analyses.

**Variables**	**Agree to get tested**		**Agree to self-isolate**		**Accept Covid-19 vaccine**	
	**if symptomatic**		**if symptomatic**		**for children**	
	**AOR (95% CI)**	***p*-Value**	**AOR (95% CI)**	***p*-Value**	**AOR (95% CI)**	***p*-Value**
**Mothers' age (years)**
<30	Reference	0.02				
≥30	2.50 (1.17–5.36)					
**Survey timing**
Before lockdown	Reference	0.006				
During lockdown	3.36 (1.41–8.02)					
**Marital status**
Other			Reference	<0.001		
Married/de-facto partner			17.15 (3.56–82.65)			
**Survey timing**
Before lockdown			Reference	0.10		
During lockdown			4.12 (0.76–22.17)			
**Fathers' education level**
Up to HSC/TAFE					Reference	<0.001
University & Higher					2.60 (1.67–4.04)	
**Survey timing**
Before lockdown					Reference	0.13
During lockdown					1.41 (0.91–2.20)	

*HSC, Higher School Certificate (Year 12); TAFE, Technical and Further Education; AOR, adjusted odds ratio, adjusted for intervention allocation, and variables in table were also adjusted to each other*.

## Discussion

This was the first Australian study on COVID-19 related study outcomes specifically involving mothers of children younger than 5 years. Since new COVID variants are likely to emerge and may have significant impacts on young children, the findings of this study are important for future public health response planning. We found that only half of the survey respondents were definitely willing to vaccinate their children against COVID-19 if a suitable vaccine becomes available. Fathers' education level was associated with mothers' willingness to have their 4 year old child vaccinated. Mothers' age or marital status is associated with their willingness to get tested or isolate if symptomatic respectively. Willingness to get tested was higher in the middle of a lockdown than prior. This suggested that mothers' willingness to get tested and self-isolate may have differed from that reported if there were no lockdown restrictions. The study findings highlighted father's education level, mother's age and marital status were related to the mother's willingness to vaccine their child and some specific mitigation measures for the COVID-19 pandemic. The lockdown factor was only associated with mothers' willingness to get tested if symptomatic.

The adverse impacts of COVID-19 infection on morbidity and mortality are higher in socially disadvantaged population groups (16). Although severe symptoms and consequences from COVID-19 in children are rare (7), there are concerns about uncertain impacts on children from the new variants. At the time of writing, Australia has already opened up the COVID-19 vaccination services to children aged 5 years and above. Scientific data on the uptake of the COVID-19 vaccine among children aged 5–11 years are not yet available. However, several studies globally have examined parents' and carers' attitudes to, and their acceptance of, vaccinating their children against COVID-19 (14, 17–19). Parents' acceptability varied, with the highest intention to vaccinate their children recorded in China (17), and South Korea (19). This high intention rate was attributed to worries about children becoming infected in the future (79%) or being quarantined if they were infected (71%). Close to 70% believed in the safety and effectiveness of the vaccines (17).

An online survey conducted across 16 countries between October and November 2020 surveyed 17,054 pregnant women and mothers of young children (18). Despite 69% of respondents overall indicating their intention to vaccinate their children, their willingness varied across countries, with acceptance levels above 85% among mothers in India, Mexico, Brazil, and Columbia, but below 52% for the US, Russia, and Australia. Mothers reported concerns about the approval of vaccines being rushed for political reasons (40%) and said they would like to see more safety and effectiveness data among children (33%) (18).

Other studies generally showed high parental hesitancy toward COVID-19 children's vaccines. A study conducted in the UK between April and May 2020 explored parents' and carers' views on the acceptability of a COVID-19 vaccine (14). It showed less than half of respondents would definitely (48%), or were leaning toward yes (41%) to accept a vaccine for their children. A study conducted in Turkey found that parental willingness for their children to receive the COVID-19 vaccine was low. The only characteristics of either parents or children found to affect parental willingness for children to receive the COVID-19 vaccine were parents working in healthcare (20). In this study, we found that fathers' education level was associated with mothers' willingness to accept COVID-19 vaccination, which could be due to the fact that higher education level parents may have more knowledge of and confidence in vaccination.

The Australian National COVID-19 Response requires that the community engage in certain behaviors such as testing and isolating when symptomatic to prevent the transmission of COVID-19 (21). Evaluating the characteristics of mothers of young children who are more or less likely to comply with this mandate is a vital aspect of our efforts for developing policy and targeted messaging to prevent the further spread of COVID-19 in vulnerable groups. Previous studies have attempted to elucidate associations between a mother's socio-demographic characteristics and their willingness to test and isolate themselves from others when they find themselves to have symptoms of COVID-19 (22, 23). For example, a survey of 167 pregnant women attending antenatal clinics in Singapore found sociodemographic factors including age >36 years, Malay ethnicity, employment in front line jobs and attendance at high-risk clinics are likely to influence the attitudes and precaution practices among pregnant women toward COVID-19 (23). That study asked mothers about their willingness to isolate after a confirmed infection with COVID-19 rather than merely being symptomatic, as in our survey. We found that mothers older than 30 years of age were more likely to get tested if symptomatic, which could be due to the factor of maturity and that they could be more responsible for their family and society Married mothers or mothers with a de facto partner were more likely to self-isolate because the husband or partner could provide more support in their isolation. Also, a single parent could experience a loss of family income due to isolation.

It is worth noting that unlike a previous survey of the same study respondents on the role of ethnicity in perceived impacts of COVID-19 (24), this survey found no associations of mothers' ethnicity with the willingness to vaccinate young children and to get tested and self-isolate. Given the global variation in willingness to vaccinate children, more qualitative research is needed to better understand the role of ethnicity in various COVID-19-related knowledge, attitudes, and practices.

### Strengths and Limitations

This survey was timely, having been conducted during the COVID-19 pandemic, and also conducted before and after an official government announcement of the Greater Sydney lockdown 2021, in which we were able to explore the potential effect of the strict lockdowns on the survey responses related to COVID-19. We used high-quality survey instruments in this study as the survey questions relating to the willingness to get tested, isolate, and vaccinate their children were based on previous publications (14, 15) and family demographic information was collected using a widely used NSW Population Health Survey Questionnaire (13). An external market survey company was contracted for this survey and interviewers had no knowledge of the specific aims of this survey.

With regards to the limitations, the cross-sectional survey design limits any causal inferences of the study findings. The survey selection bias was evident as the survey used a convenience sample and survey respondents participated in an existing intervention trial. However, efforts were made to minimize potential intervention effects by adjusting for group allocation in data analyses. Due to the limited length of the questionnaire, we were unable to collect data on mothers' health conditions and their knowledge and understanding of COVID-19 prevention measures and vaccines which could be confounding factors. Additionally, we acknowledge that the generalizability of the survey findings could be limited due to the location where the survey was conducted. Further qualitative research is required to understand why specific family demographics were related to mothers' willingness to vaccinate their young children or to be self-isolated and tested if symptomatic.

## Conclusion

The COVID-19 prevention strategies require community actions that include testing and isolation if they have COVID-19 symptoms as well as vaccinating children if vaccines are available. Although willingness to be tested and isolated if symptomatic was high across all families, mothers' age and marital status were associated with testing and isolation respectively. Fathers' education level was associated with mothers' willingness to vaccinate their young children if a suitable vaccine becomes available. There is a great need to take into account specific family demographics for the promotion of prevention strategies for tackling the COVID-19 pandemic.

## Data Availability Statement

The raw data supporting the conclusions of this article will be made available by the authors, without undue reservation.

## Ethics Statement

The studies involving human participants were reviewed and approved by the Ethics Review Committee of Sydney Local Health District (Protocol No. X16–0360 and LNR/16/RPAH/495 and Protocol No. X18–0387 and HREC/18/RPAH/545). The patients/participants provided their written informed consent to participate in this study.

## Author Contributions

LW contributed to study conception and design, data interpretation, and preparing the first draft. HX contributed to data analysis, interpretation, and presentation. EK, LB, and RC contributed to the literature search. All authors contributed to project implementation of the existing trial, provided critical comments on the drafts, and approved the final version of the paper. All authors contributed to the article and approved the submitted version.

## Funding

This study was part of an intervention trial funded by the NSW Health Translational Research Grant Scheme 2016 (ID number: TRGS 200) and the Australian National Health and Medical Research Council Partnership Project APP1169823.

## Conflict of Interest

The authors declare that the research was conducted in the absence of any commercial or financial relationships that could be construed as a potential conflict of interest.

## Publisher's Note

All claims expressed in this article are solely those of the authors and do not necessarily represent those of their affiliated organizations, or those of the publisher, the editors and the reviewers. Any product that may be evaluated in this article, or claim that may be made by its manufacturer, is not guaranteed or endorsed by the publisher.

## References

[1] WHO. Coronavirus (COVID-19) Dashboard. Available online at: https://covid19.who.int/ (accessed January 8, 2022).

[2] Australian Government Department of Health. Coronavirus (COVID-19) at a glance–19 January 2022. Available online at: https://www.health.gov.au/resources/publications/coronavirus-covid-19-at-a-glance-19-january-2022 (accessed January 8 2022).

[3] Australia Government Department of Health. Available online at: https://www.health.gov.au/initiatives-and-programs/covid-19-vaccines (accessed January 8, 2022).

[4] MacDonald NE SAGE SAGE Working Group on Vaccine Hesitancy. Vaccine hesitancy: definition, scope and determinants. Vaccine. (2015) 33:4161–4. 10.1016/j.vaccine.2015.04.036 25896383

[5] Aw J Seng JJB Seah SSY Low LL. COVID-19 vaccine hesitancy-a scoping review of literature in high-income countries. Vaccines. (2021) 9:900. 10.3390/vaccines9080900 34452026 PMC8402587

[6] Cascini F Pantovic A Al-Ajlouni Y Failla G Ricciardi W. Attitudes, acceptance and hesitancy among the general population worldwide to receive the COVID-19 vaccines and their contributing factors: a systematic review. EClinicalMedicine. (2021) 40:101113. 10.1016/j.eclinm.2021.101113 34490416 PMC8411034

[7] Williams P Koirala A Saravanos G Lopez L Glover C Sharma K. COVID-19 in children in NSW, Australia, during the 2021 delta outbreak: severity and disease spectrum. medRxiv. (2021). 10.1101/2021.12.27.21268348

[8] Wen LM Rissel C Baur LA Hayes AJ Xu H Whelan A. A 3-arm randomised controlled trial of communicating healthy beginnings advice by telephone (CHAT) to mothers with infants to prevent childhood obesity. BMC Public Health. (2017) 17:79. 10.1186/s12889-016-4005-x 28088203 PMC5237545

[9] Wen LM Rissel C Xu H Taki S Smith W Bedford K. Linking two randomised controlled trials for Healthy Beginnings©: optimising early obesity prevention programs for children under 3 years. BMC Public Health. (2019) 19:739. 10.1186/s12889-019-7058-9 31196026 PMC6567558

[10] Ekambareshwar M Mihrshahi S Wen LM Taki S Bennett G Baur LA. Facilitators and challenges in recruiting pregnant women to an infant obesity prevention programme delivered *via* telephone calls or text messages. Trials. (2018) 19:494. 10.1186/s13063-018-2871-5 30219067 PMC6139132

[11] Wen LM Rissel C Xu H Taki S Buchanan L Bedford K. Effects of telephone support and short message service on infant feeding practices, 'tummy time' and screen time at 6 and 12 months of child age: a 3-arm randomized controlled. JAMA Pediatr. (2020) 174:657–64. 10.1001/jamapediatrics.2020.0215 32282034 PMC7154951

[12] Wen LM Xu H Taki S Buchanan L Rissel C Phongsavan P. Effects of telephone support or short message service on body mass index, eating and screen time behaviours of children age 2 years: a 3-arm randomized controlled trial. Pediatr Obes. (2022) 17:e12875. 10.1111/ijpo.12875 34821063 PMC9285384

[13] Centre for Epidemiology Research NSW NSW Department of Health. New South Wales Adult Health Survey. (2017). Available online at: https://www.health.nsw.gov.au/surveys/adult/Documents/questionnaire-2017.pdf (accessed January 8, 2022).

[14] Bell S Clarke R Mounier-Jack S Walker JL Paterson P. Parents' and guardians' views on the acceptability of a future COVID-19 vaccine: a multi-methods study in England. Vaccine. (2020) 38:7789–98. 10.1016/j.vaccine.2020.10.027 33109389 PMC7569401

[15] Bonner C Batcup C Ayre J Pickles K Cvejic E Copp T. Behavioural barriers to COVID-19 testing in Australia. medRxiv. (2020). 10.1101/2020.09.24.20201236

[16] Hawkins RB Charles EJ Mehaffey JH. Socio-economic status and COVID-19-related cases and fatalities. Public Health. (2020) 189:129–34. 10.1016/j.puhe.2020.09.016 33227595 PMC7568122

[17] Wan X Huang H Shang J Xie Z Jia R Lu G. Willingness and influential factors of parents of 3-6-year-old children to vaccinate their children with the COVID-19 vaccine in China. Hum Vaccin Immunother. (2021) 17:3969–74. 10.1080/21645515.2021.1955606 34344258 PMC8828112

[18] Skjefte M Ngirbabul M Akeju O Escudero D Hernandez-Diaz S Wyszynski DF. COVID-19 vaccine acceptance among pregnant women and mothers of young children: results of a survey in 16 countries. Eur J Epidemiol. (2021) 36:197–211. 10.1007/s10654-021-00728-6 33649879 PMC7920402

[19] Choi SH Jo YH Jo KJ Park SE. Pediatric and parents' attitudes towards COVID-19 vaccines and intention to vaccinate for children. J Korean Med Sci. (2021) 36:e227. 10.3346/jkms.2021.36.e227 34402237 PMC8352785

[20] Yilmaz M Sahin MK. Parents' willingness and attitudes concerning the COVID-19 vaccine: a cross-sectional study. Int J Clin Pract. (2021) 75:e14364. 10.1111/ijcp.14364 33998108 PMC8236907

[21] Australian Government National Plan to transition Australia's National COVID-19 Response. Available online at: https://www.pm.gov.au/sites/default/files/media/national-plan-to-transition-australias-national-covid-19-response-30-july-2021.pdf (accessed January 9, 2022).

[22] Carlsen E Ø Caspersen IH Trogstad L Gjessing HK Magnus P. Public adherence to governmental recommendations regarding quarantine and testing for COVID-19 in two Norwegian cohorts. Preprints from medRxiv and bioRxiv. (2020). 10.1101/2020.12.18.20248405

[23] Lee RWK Loy SL Yang L Chan JKY Tan LK. Attitudes and precaution practices towards COVID-19 among pregnant women in Singapore: a cross-sectional survey. BMC Pregnancy Childbirth. (2020) 20:675. 10.1186/s12884-020-03378-w 33167918 PMC7652671

[24] Wen LM Xu H Jawad D Buchanan L Rissel C Phongsavan P. Ethnicity matters in perceived impacts and information sources of COVID-19 among mothers with young children in Australia: a cross-sectional study. BMJ Open. (2021) 11:e050557. 10.1136/bmjopen-2021-050557 34824114 PMC8627368

